# Intergovernmentalism and the crisis of representative democracy: The case for creating a system of horizontally expanded and overlapping national democracies

**DOI:** 10.1177/13540661221106909

**Published:** 2022-07-01

**Authors:** Joachim Blatter, Johannes Schulz

**Affiliations:** University of Lucerne, Switzerland

**Keywords:** European Union, democratic deficit, transnational democracy, populism, technocracy, multiple citizenship

## Abstract

Technocratic intergovernmentalism has undermined the preconditions for its own success as a democratic project of transnational cooperation. It has triggered populist reactions within nation states and helped to discredit the intermediary institutions (parties and parliaments) that connect democratic will-formation and joint decision-making. This rise of populism and its alignment with nationalism, in consequence, hampers joint decision-making in the international realm. We argue that representative democracies can overcome the negative spiral between technocratic intergovernmentalism and nationalist populism by mutually granting their citizens the right to elect representatives not only in their domestic parliament, but also in the parliaments of ‘consociated democracies’. Such a system of horizontally expanded and overlapping national democracies can serve three functions: it re-empowers citizens in a world of cross-border flows, it curbs the self-destructive polarization of party systems and it facilitates cooperation among democracies within the European Union (EU) and beyond. Finally, we discuss three competing approaches: Liberal Multilateralism, Deliberative Transnationalism and Republican Intergovernmentalism. We point to common ground, but also show how our approach avoids their main pitfalls.

## Introduction

Liberal, representative democracies have spearheaded the creation of a system of international governance that stimulates and/or regulates cross-border flows of information, financial and human capital, goods and ‘bads’ (e.g. pollution, crime, viruses). The dominant operative mode in this system is an intergovernmentalist one, epitomized by major political decisions made at international summits among national executives. While such decisions are made behind closed doors, they are performed and presented as dramas, with national executives playing the lead roles of warriors for the national interest. Furthermore, important competences are delegated to experts and technocratic institutions like the International Monetary Fund or the European Central Bank, which are neither accountable to individual citizens nor to their representatives.

The feeling of disempowerment and alienation that this has caused, especially among ‘globalization losers’, has, arguably, played an important role in preparing the ground for the success of populist demagogues, who pit themselves as the leaders of a heroic effort to help the ‘ordinary (sedentary/native) people’ throw of the shackles of ‘oppression’ by ‘transnational elites’ (e.g. Bickerton and Accetti, 2017). It is unsurprising then, that international agreements and supranational institutions like the European Union (EU) have increasingly come under attack by nationalist populist forces. Not only Brexit but the growing arduousness of finding common ground within the EU indicates that technocratic intergovernmentalism has undermined its own precondition for success: an inter-democratic order can only function if national constituencies acknowledge that their representatives have to respect the perspectives and interests of other people(s), not only when they are involved in joint decision-making on the international level, but also when they decide autonomously about policies with strong external effects.

Importantly, technocratic intergovernmentalism has not only undermined its precondition for successful international cooperation and regional integration. It has also caused significant collateral damage to the democratic process within member states. Representative democracy is increasingly caught between the Scylla of technocratic intergovernmentalism and the Charybdis of populist politics in the national realm: both are ‘disfigurations’ (Urbinati, 2014) of the process of democratic opinion and will-formation that weaken the influence of important intermediary organizations like parties and parliaments and contribute to the undermining of democracies’ commitment to political and cultural pluralism (Caramani, 2017).

In this paper, we propose that democracies jointly re-constitutionalize what Mark Warren calls the first function of democracy (‘empowered inclusion’) in order to avoid the further disfiguration of democratic will-formation and deterioration of the preconditions for positive and negative forms of integration. We propose that they give citizens of participating states the right to elect a (limited) number of ‘consociated representatives’ into the parliaments of consociated states. The main idea is to channel popular dissatisfaction into productive lines. Rather than seeing no other option but to strengthen populist nationalists at home, citizens can use their additional voting rights to make their voices heard in foreign places of decision-making they perceive to have significant impact on their life. If Italy and Germany, say, where to sign such a joint declaration, the citizens of Italy would be able to elect representatives into the German parliament and vice versa, giving them, or this is the idea, back some control over transnational issues that systematically affect them. What is more, negotiation concerning international cooperation would be brought out from the closed chambers of international summits and technocratic institutions and into the open halls of state parliaments and the public debates surrounding electoral campaigns.

There are many causes for the rise of technocracy, plebiscitarianism and populism and we do not claim to address all or even most of them. Our focus, however, on political institutions and our attempt to re-embed intergovernmental decision-making in transnationalized forms of collective opinion and will-formation has three important advantages: first, it builds on domestic democratic structures and processes that have firmly established themselves as the core sites of opinion formation and decision-making, rather than a protracted and difficult transfer of competencies and identities from the national to the inter- or supranational level. A horizontal expansion of representative democracy may, second, increase not only the legitimacy, but also the efficiency of (joint and formally autonomous) rulemaking among democracies. This may play a role in strengthening democracies’ defences, internally, against a populism that pushes it towards a slippery slope that tilts towards authoritarianism (see, e.g. Dresden and Morjé Howard, 2016) and, externally, against authoritarian competitors like Russia or China. Finally, a horizontal expansion of national democracy does not imply ‘lighter’ or ‘thinner’ forms of democracy and citizenship, typical of post-national or federalist proposals, but a multiplication of the core mechanisms that link the ruled and the rulers in a representative form of democracy: membership and voting rights plus elections and the resulting partisan/parliamentarian representation.

In the first part of this paper, we show how an intergovernmentalist system of regulation and international cooperation, with its very limited forms of empowered inclusion, contributes to the most important disfigurations that democracies are currently faced with: technocracy, plebiscitarianism, populism and a crisis of the (traditional) political party. In the second part of the paper, we present our proposal which asks democratic state-peoples and their individual members to jointly create a system of horizontally expanded and overlapping state demoi/electorates.^1^ The explicit constitutionalization of a system of overlapping state electorates, which already emerges as a side-effect of spreading dual citizenship and external voting, provides the groundwork for expanding and strengthening national parliaments as well as national parties. The transnationalization and strengthening of these important intermediary institutions helps to refigure democratic will-formation and joint decision-making. Furthermore, individual citizens are empowered as agents of transnational politics: they are provided with the opportunity to trace structures of cross-border influence and domination and to shape opinion formation and decision-making in those sites of political power they perceive as having a significant impact on their lives. In the final part of this paper, we compare our proposal to three influential alternatives for reconfiguring transnational democracy: Deliberative Transnationalism (DT), Republican Intergovernmentalism (RI), and Liberal Multilateralism (LM). These alternative approaches act as a foil that allows us to further clarify and defend our proposal.^2^

## Intergovernmentalism and its discontents

We ground our argumentation on Mark Warren’s account of democratic theory. Warren (2017) argues that functioning democracies have to meet three requirements: they must ‘include those people entitled to voice and impact into political processes through distributed empowerments’; they ‘need to form this input into collective agenda or wills’; and finally, they ‘need to make decisions through which “the people” are reflexively constituted into collective agents capable of doing collective things for themselves’ (p. 43). We draw on Warren here, partly because his model of democracy elegantly and plausibly blends the deliberative and aggregative strands of democratic theory that have come to dominate the field. It does so, more importantly, in a way that draws our attention to the importance of empowered inclusion (the first requirement) as the fundamental precondition for meeting the two other requirements: will-formation and decision-making. This also means, and this is where our analysis begins, that pathologies in empowered inclusion will automatically lead to pathologies in will-formation and decision-making. In the following, we show that intergovernmentalism meets the first requirement in a particular – bifurcated – way, and that this has problematic consequences for our collective ability to meet requirements two and three.

Democratic Intergovernmentalism (henceforth ‘Intergovernmentalism’), as a system of regulating cross-border (inter)dependencies and fostering political integration among democratic states, operates on the basis of a division between the domestic and international realm of politics. In each of these realms a different kind of actor – individual people (citizens) in the domestic sphere and state-peoples (nations) in the international sphere – is recognized as the primary principal and empowered through voice and vote. This particular form of inclusion gives state governments a central role in international politics to the detriment of processes of will-formation and collective decision-making. First, it allows them to inverse the correct temporal order of democratic processes in which opinion formation should precede decision-making. Second, it contributes to all the ‘disfigured’ ways of opinion formation that plague present-day democracies: technocracy, plebiscitarianism and populism. Finally, it poses a fundamental dilemma for political parties, which are simultaneously expected to function as intermediary organizations that are responsive to their voters and, when in government, as responsible partners in international cooperation.

### Bifurcated realms, one-dimensional inclusions and disordered functions

An intergovernmental approach to dealing with cross-border flows and transnational (inter)dependencies operates on the presumption that politically the domestic realm and the international realm are clearly separated. The governments of peoples who are recognized as sovereign nation states are the only actors who play important roles in both arenas: in the domestic realm, they function as agents who are instrumental for fulfilling the will of state-peoples. In the international realm, they function as representatives of their state-peoples in international interactions. In consequence, ‘intergovernmentalism’ not only gives a powerful gate-keeper position to state governments, it also fulfils the inclusion function of a democratic order in a bifurcated way: within the domestic realm, only members of the national community are included into its demos and empowered as citizens; members of other nation states are neither included nor politically empowered. In the international realm, only state-peoples and their governmental representatives are recognized as principals of the international community; individual citizens are neither included nor empowered. The latter are only indirectly included as members of their nations and only represented collectively through their state governments.

In sum, intergovernmentalism is based on one-dimensional answers in respect to which kind of actors must be included and empowered in the two realms. A first problematic consequence is that this reverses the order in which collective will-formation and decision-making should take place. From a normative point of view, domestic deliberation and will-formation must come before international decision- and rulemaking. In intergovernmental practice, this order is often reversed. Intergovernmental negotiation and decision-making at international summits and in supranational institutions and bodies often precede domestic opinion and will-formation, which sees individual citizens and their parliamentary representatives reduced to spectators that can only approve or reject what their government has negotiated with other governments. This is problematic not only because it signifies a failure in empowered inclusion of individuals, but because it reverses the correct functional order between opinion formation and decision-making. Opinion formation through deliberation within the public sphere, political parties and parliaments must take place prior to collective decision-making. Only then can policy-makers choose from adequately formulated alternative options, which reflect the voices and perspectives channelled in processes of opinion formation (Warren, 2017: 43). Such a disordered process contributes to what Nadja Urbinati (2014) calls ‘disfigured’ forms of opinion formation.

### Disfigured opinion formation: technocracy, plebiscitarianism, populism

Nadja Urbinati (2014: 8–9) argues that technocracy, plebiscitarianism and populism are disfigured forms of opinion formation because they radicalize either the cognitive/epistemic, aesthetic/symbolic or political aspect of opinion formation to the detriment of the two other aspects. In doing so, they undermine political pluralism and a proceduralist understanding of democracy. In the following, we briefly sum up how intergovernmentalism contributes to these disfigured forms of opinion formation.

The technocratic form of disfigured opinion formation divorces democracy of its political nature by making it a process of achieving the ‘correct outcome’ (Urbinati, 2014: 7). Technocrats believe that politics needs to be, as Urbinati puts it, purified of ideological competition and the cognitive biases of the electorate. This kind of reasoning contributes to the growing importance of the executive branch of government, to an increasing reliance on experts, and to the rise of regulatory agencies (e.g. central banks) that are not directly accountable to the people or their representatives.

Intergovernmentalism is not only expressive of the technocratic logic, it adds historical and functional justifications for the de-politicization of collective will-formation. The intergovernmentalist system has its genesis in the negotiations of political elites that operated under the shock of the Second World War, the Nazi regime and Stalinism. Technocratic forms of political integration in Europe were established to reduce the risk of another fatal outbreak of popular emotions aligned with (competing) nationalistic ideologies (Biebricher, 2020). A broad spectrum of technocratic instruments and institutions has not only evolved in response to historical catastrophes, but also in order to address the functional need to overcome different national interests in a consensus-based system: the intensive preparation of political summits in administrative committees (what is called ‘comitology’ in the EU context, e.g. Joerges and Neyer, 1997), the involvement of so-called ‘epistemic communities’ and the reliance on their scientific reputation (Haas, 1992), the spread of ‘performance indicators’ (Kelley and Simmons, 2019) and the delegation of tasks to international regulatory agencies which are not directly accountable to parliamentarian representatives (Hooghe and Marks, 2015) are some of the most important ones.

The second form of disfiguration that Urbinati (2014: 171–227) describes is a closely related but analytically distinct form of de-politicization. In a *plebiscitarian* democracy, political leaders perform politics as a theatrical spectacle, the citizens are reduced to the role of the spectator who voices their approval to political decisions that were made without their input. Intermediary organizations like political parties are misused for the organization of public approval. Urbinati identifies the rise of visual mass media as a main driver for the plebiscitarian disfiguration of opinion formation and for the (re-)personalization of politics.

Intergovernmentalism has contributed significantly to the rise of plebiscitarian disfigurations of democracy. A central characteristic of intergovernmentalism is the pooling of political sovereignty and regulatory competences to intergovernmental institutions like the European Council. Rather than delegating competences to new regulatory agencies, such pooling sees state governments give up their right to regulate a policy field autonomously (Hooghe and Marks, 2015). Not only the creation, but also a later change of these regulations depends on finding a consensus or a majority among state governments. Like delegation to regulatory agencies, pooling of competences strengthens technocratic forms of opinion formation, since the joint decisions must be prepared and facilitated with the help of policy experts and bureaucrats. It also contributes to the rise of plebiscitarianism, however, because it creates a specific kind of forum and one that shapes the public’s perception of the political process. International summits, especially those that see governmental leaders meet, are often portrayed, especially in the mass media, as the heart of the political process. Leadership summits are particularly attractive to the media who can portray politics as a drama in which national leaders perform spectacular roles. The public is allowed only glimpses of this drama and their representatives are left with the task of approving (or, in rare cases, disapproving) of the agreements that the leaders of the executive have negotiated for behind closed doors.

Most importantly, intergovernmentalism contributes to a third democratic disfiguration: populism (Urbinati, 2014: 128–170). As Mudde and Kaltwasser (2017) have plausibly argued, populist ideologies pit the ‘morally pure people’ against the ‘corrupt elites’. Populism is complementary to plebiscitarianism (the populist leader claims to speak to and for the people who are expected to do no more than voice their approval) but populism and technocracy are functional opposites. Technocrats try to circumvent politics in an effort at avoiding the necessary over-simplifications that take place in a political discourse. Populists, on the other hand, claim to act as a corrective that redeems the promise of popular sovereignty against an overly rigid, pragmatic and elitist political apparatus (Canovan, 1999). Intergovernmentalism play a crucial role in the mutual radicalization of technocracy and populism since it relegates the two forms of will-formation into distinct realms. As we argued before, the intergovernmental approach to international cooperation and regional integration lends itself towards a technocratic form of will-formation in the international realm. Within the domestic realm, it is complemented by forms of plebiscitarianism which see state-peoples acclaim the performances of their governmental leaders in struggling for the national interest. Plebiscitarian technocracy was largely accepted by state-peoples, as long as the perception prevailed that all profit from international cooperation and that governmental leaders successfully defend a unified and homogeneous national interest. The belief that everyone profits from a common currency or free trade agreements has been undermined, however, by developments like the ‘Euro crises’, and the ‘China shock’ (e.g. Autor et al., 2013; Guiso et al., 2019).

This triggered populist reactions in the domestic realm, only furthered by established parties, that, in trying to cater to the median voter, stubbornly clung to an increasingly questionable narrative of mutual gains and coherent national interest. Populist leaders used the opportunity to exploit and exaggerate the obvious tensions in these narratives. Rather than acknowledging the plurality of interests within and across state-people, which make transnational cooperation a complex endeavour, populists, on the left and the right, portray international negotiation and compromise-building as the treason of elites against people whose interests they no longer represent. Three aspects of intergovernmentalism in particular allowed the treason narrative to gain traction. First, its technocratic nature undermines the institutional links between the rulers and the ruled. Second, its transnational nature distorts the imagined congruence between the rulers (the national government) and the ruled (the nation). Both lead to a feeling of alienation among the people and to a sense of loss of control. Finally, the (neo)-liberal bias which prefers ‘negative integration’ (regulatory competition and de-regulation through mutual acceptance of state regulations) over ‘positive integration’ (re-regulation through a commonly agreed regulation; Scharpf, 1999) undermined the capacities of nation states to shield their citizens against the disruptive forces of the global market and to support them in coping with socio-economic and technical (e.g. digital) transformations (see Ibsen, 2019).^3^

Both this (neo)-liberal bias and the growing feeling of alienation give populists the opportunity to portray governmental leaders and their advising experts as part of a ‘corrupt elite’ that abuses intergovernmental institutions and networks to collude in their own interest and according to their own values. This to the detriment of a people who only stand to lose from cross-border flows of capital and goods (left-wing populism), and whose culture and values are endangered by migrants or neglected by mobile people (right-wing populism). Populists argue that the only alternative to the rule of the corrupt elites is that state-peoples act unilaterally to ‘take back control’. This is a stance that leads to a democratic disfiguration of both domestic and transnational politics: domestically, it leads to discourses and policies of exclusion as well as an attack on intermediary institutions such as parliaments and parties (who are increasingly thought to be in bed with governmental leaders). Internationally, it leads to significant difficulties in cooperating with other state-peoples.

### Distorted political parties and party systems: torn apart between responsiveness and responsibility

Political parties constitute a crucial intermediary institution that connects the ruled to the rulers. In contrast to other important intermediary institutions like interest organizations and the media, they fulfil important roles both within democratic processes of will-formation and within democratic procedures of decision-making. In the former, they pick up or mobilize citizens’ perspectives and interests and bundle them into a plurality of divergent, more or less ideologically coherent policy proposals and programmes (making meaningful choices possible). In the latter, they organize legislative majorities and – crucially – recruit and train members of government.

Today, political parties, particularly in Western Europe, face a crisis that is – at least partly – linked to the intergovernmentalist system and the technocratic, populist and plebiscitarian disfigurations that become increasingly significant in its wake. In a process that Katz and Mair (2009) describe as the ‘cartelization’ of party democracy, established parties have turned away from contentious political positions and handed responsibility for difficult decisions to expertocratic institutions in reaction not only to a drastic fall in social support but to the functional pressures of the intergovernmentalist system: European integration after the end of the Soviet Union was driven by the conviction that in a world of liberalized cross-border flows, core policies (e.g. monetary policy with its impact on inflation and unemployment) must be delegated to technocratic institutions in order to shield them from partisan competition and its resulting unpredictability. This process of de-politicization further alienated voters, though, and drove them into the arms of populist demagogues. The rise of populist parties has led to an epochal shift in the party system, seeing the birth of a new cleavage that pits advocates of domestic pluralism and international cooperation against defenders of externally closed and internally coherent, competing nation states against each other (Kriesi et al., 2012).

Faced with the double pressure of the demands of an intergovernmentalist system aimed at international cooperation and populist parties and voters that publicly push demands for a re-nationalization of the political system, established parties are confronted with a dilemma. They find themselves torn between ‘responsiveness’ towards the increasingly nationalist demands of their voters and ‘responsible’ governance,^4^ which copes with the reality of an increasingly interconnected world primarily by setting up and cooperating within intergovernmental institutions. The somewhat schizophrenic solution that many parties seem to have found in dealing with this dilemma is a shift in behaviour and communication depending on the *mode* they find themselves in: election/campaign mode or governance mode. In the election/campaign mode they have strong incentives to cater to the national interests of those who constitute its own demos, perhaps even to portray an ‘external other’ as a threat to national identity, which is often a viable strategy for winning elections. In governance mode, on the other hand, the very same parties have to create joint regulations within a system of intergovernmental decision-making, striving for ‘responsible’ policy-making, in the sense of having to take into account perspectives and interests that go beyond those of their electorate. While some parties will choose the ‘responsiveness’ horn of the dilemma and stress their efforts to act in the national interest (populist parties in particular) and others will turn towards the ‘responsibility’ horn of the dilemma (traditional ‘cartel’ parties in particular), the entire party system is faced with the same dilemma and one that threatens party democracy as we know it.

The responsibility–responsiveness dilemma that traditional parties are faced with in an intergovernmentalist system acts like grist to the mills of populist rhetoricians, who are given the opportunity to push their claim that it is not only the executive but the representatives of traditional parties that ‘collude’ to betray the interests of the people. In turn, established parties, accused of failing to be *responsive* to the electorate, accuse the populist newcomers of failing to be *responsible*: first, as agents of liberal-democracy and its principle of pluralist inclusion, second, as agents of an international cooperative order that requires negotiation, compromise and an adherence to past agreements. Increasingly, these two sides of the party spectrum interact in a way that betrays a Kelsenite understanding of democracy as a game of parliamentary competition between adversaries and sees it slide into a Schmittian realm of politics as a conflict between enemies (Wolkenstein, 2019). Parliamentary politics works as long as parties view their interaction as a competition between policy proposals and programmes, where all competing parties are oriented towards fulfilling the common interest but disagree on how exactly to get there (White and Ypi, 2016). In a hyper-politicized party system, the other is no longer presented as a competitor but an immoral enemy who no longer has the common interest in mind. The turn from ‘agonism’ to ‘antagonism’ (Mouffe, 1999) has disfiguring and destabilizing effects not only on domestic democratic systems however. It also creates serious difficulty for attempts at advancing international cooperation and problem-solving capacities when the antagonistic style of politics spills over into the international realm.

## Expanding state-peoples, parliaments and parties horizontally: a proposal

We propose that in order to address the diagnosed problematic consequences of technocratic intergovernmentalism, representative democracies should join forces and create a system of horizontally expanded and overlapping state demoi, parliaments and parties. In this section, we briefly present the major elements of our proposal and lay out how the proposed reforms might tackle the diagnosed disconnections, disfigurations and distortions in a way that simultaneously considers the need to democratize *and* the need to facilitate the cooperation among representative democracies. We start with describing how state-peoples and individual people jointly constitutionalize an already emerging system of horizontally overlapping electorates. Furthermore, we describe how such new forms of empowered inclusion do not only help to refigure democratic will-formation and joint decision-making, but also to strengthen the two core intermediary institutions that link these two functions: parliaments and parties.

We believe that the proposal is particularly well suited for the member states of the European Union, but it could also be set up (first) among two countries like France and Germany or by liberal democracies beyond Europe (e.g. Canada and the United States). The only principled preconditions are that the participating state-peoples share a fundamental commitment to representative democracy and that they have established the corresponding institutions domestically.

### Reconnecting the international and domestic realms by jointly constitutionalizing ‘consociations of democratic state-peoples’ and including ‘consociated citizens’

Representatives of democratic state-peoples (ideally the head of government and the head of parliament) should sign a ‘joint declaration of interdependence and identification’, thus forming a ‘consociation of democratic state-peoples’^5^ and expressing their dual commitment to democratize and to strengthen their joint rulemaking (presuming that the latter is predominantly characterized by the pooling of sovereignty and competences, and not by transferring it to a higher level). The participating state-peoples ratify the joint declaration (ideally through a referendum, or in accordance with the established procedures for major international treaties) and thereby identify each other as collective members of a consociated community of liberal democracies. On the basis of such mutual recognition, each state-people offers individual members of the other state-peoples the status of ‘consociated citizen’.

Citizens of participating states are given the opportunity to sign ‘declarations of interest and identification’ on a regular basis (e.g. in combination with the national elections in their state). These declarations see them assert their legitimate interest in participating in the will-formation and decision-making processes of one or more of the consociated state(s) because they are ‘subjected to’ joint regulations *and* because they are systematically affected by the policies of the other state(s) – the interest part of the declaration. At the same time, they explicitly recognize that the consociated state(s) has/have become a legitimate part of their overall system of governance, that they will obey the rules produced within this interdependent system and that they feel responsible for the functioning of the system – the ‘identification part’ of the declaration. By signing this declaration, these citizens register (for a specified time, e.g. one or two electoral periods) as ‘consociated citizens’ in the corresponding consociated state(s). This citizenship status comes with the right to vote (a limited number of ‘consociated representatives’, see below) and with the right to stand as a candidate in the national election of the consociated state.

What we propose is that democratic state-peoples and their individual members reconnect the international and domestic realms by jointly and explicitly constitutionalizing a system of horizontally overlapping state demoi. This sees them overcome the bifurcated forms of empowered inclusion that characterizes intergovernmentalism – not by including individual citizens in the international realm, as it is often proposed and already institutionalized in the EU, but by including citizens in multiple domestic realms. Furthermore, they draw on a system that is already emerging in an almost unnoticed, non-reflected and uncoordinated way: the growing acceptance of multiple citizenships by states together with the trend to grant voting rights to non-national residents and to non-resident nationals already leads to a situation in which a growing number of citizens – but not all – can vote in more than one state (Bauböck, 2005; Blatter, 2011; Blatter et al., 2022).^6^

State-peoples function as a *pouvoir constituant* in this process. Their power lies in making the founding decision to join the consociation of democratic state-peoples and in negotiating the basic framework that regulates the simultaneous expansion of state electorates, parliaments and parties. Nevertheless, individual citizens are given the opportunity to trace transnational power structures and to contribute to realigning *demoi* and *kratoi* in a system of governance that is characterized by a mix of positive integration (cooperative rule-making) and negative integration (competitive rule-making), which often sees less powerful countries forced to adjust to more powerful countries.

The co-constitutive role of (particular groups of) individual citizens and collective state-peoples is particularly visible in the way we envision the implementation of our proposal in a multilateral setting (e.g. within the EU). In addition to limiting the number of consociated representatives that can be elected into a foreign parliament (which is already the case in a bilateral setting), an implementation in a multilateral setting demands that the number of member states towards which consociated citizens can send consociated representatives must be limited as well. This ensures that an overburdening of individuals and overcrowding of state parliaments is avoided. To make this more concrete, the idea is that domestic election ballots will provide citizens with the opportunity to choose a maximum of, say, five countries (if this is the limit the multilateral agreement has fixed) in which they would like to register as consociated citizens. It is only the five states most commonly chosen by the citizens that consociated representatives will be sent to. This first step represents a regularly occurring act of re-constituting *demoi*, in which citizens trace currently existing transnational power structures (as they perceive them), and, in doing so, realign *demos* and *kratos* in their collective form as state-peoples. In contrast, in a second step, which sees citizens enact their electoral rights as consociated citizens, they act as individual members of interest groups.

Our proposal envisions, for multilateral settings, that is, the enactment of ‘diffuse’ rather than ‘specific’ reciprocity. This has important consequences, in particular the fact that states which are perceived as particularly powerful (e.g. Germany or France) have to accept that they will have to allow for the establishment of far more seats for consociated representatives than they can send to the parliaments of states perceived as weaker (e.g. Estonia or Portugal). From a normative point of view, this is desirable, since it corresponds to the idea that the re-constitution of *demoi* is a re-occurring act of aligning *demoi* to *kratoi*. In political practice, powerful states might accept such an asymmetry, first because the consociated representatives function as ‘warning systems’ that help them better navigate a system of joint and interdependent governance (Schmitter, 1997) and second, because it provides a certain legitimacy to the dominant or even hegemonial positions countries like Germany and France take up as a consequence of their socio-economic power.

### Refiguring democratic will-formation and facilitating joint decision-making by empowering and expanding state parliaments simultaneously

Such an expansion of nation state parliaments through the inclusion of consociated representatives makes the empowerment of national parliaments vis-à-vis state governments in international affairs much less risky for joint decision-making and more productive for democratic will-formation. The empowerment of parliaments can take place along two principled ways, both of which aim to curtail the gate-keeper position of state governments. First, national parliaments forge their own international networks and platforms. These pluralist parliamentarian networks and platforms complement the bureaucratic and technocratic transnational networks dominated by executives and experts and thereby rebalance and refigure opinion formation in the international realm. Second, state parliaments get – individually in the domestic realm and jointly on the international/EU level – initiating and veto powers in international affairs. Their empowerment within states gives them greater influence over the agendas and positions of their governments. Their empowerment across states, on the other hand, gives them greater influence on agenda-setting and negotiation on the international/European level. This increase in parliamentary powers counteracts the drift towards plebiscitarianism that comes with intergovernmentalism since it reduces the opportunities of governmental leaders to stage themselves as the sole defenders of the national interest and helps to rearrange an adequate normative order in democratic rulemaking in which pluralist will-formation takes place prior to joint decision-making.

We also argue, however, that an empowerment of state parliaments has to go hand in hand with a horizontal expansion of these parliaments. Such an expansion allows ‘consociated representatives’ to bring the perspectives and interests of consociated citizens into the domestic will-formation and decision-making processes of the consociated states. Thus, the articulation of perspectives and positions of other state-peoples are no longer restricted to meetings that take place behind closed doors in international summits and their preparatory proceedings (or taken into account by international courts), but presented in the domestic agora of representative democracies. This gives transnational politics a much-needed element of transparency and makes it less vulnerable to a generic kind of criticism often pushed by populist agents who portray transnational cooperation as a collusion between elites that can never be democratically legitimate. This, in turn, makes it easier for states representatives to openly pursue compromising policies in international negotiations.

Even more importantly, the horizontal expansion of parliaments gives a boost to popular sovereignty, that is, to the empowered inclusion of individual citizens. As laid out in our problem diagnosis, executive intergovernmentalism has contributed to a shift in the way in which citizens can articulate political dissatisfaction. The more governing parties are forced to follow ‘responsible’ policies that emerge out of transnational expert networks, the less voting for alternative traditional parties seems to make a difference. The dissatisfaction that results from this perceived disempowerment is articulated in votes for populist parties (where this expresses a general distrust towards colluding elites and distant experts), or by voting for nationalist parties (where this expresses a distrust of state representatives who are seen to compromise national interests in international negotiations). To make matters worse, populism and nationalism tend to align, disfiguring democratic will formation and disrupting international decision-making simultaneously (Brubaker, 2017).

In a system of overlapping state electorates and transnational elections, dissatisfied citizens gain new and promising alternatives for making their voices heard. First, the mere act of registering as a consociated citizen in a consociated state itself has an important expressive function: it sends the message that the consociated state’s actions are seen to significantly affect the life of citizens in other nation-states. Take, for example, Greek or Italian citizens who have come to believe that the German or Dutch governments are the main culprit in installing austerity politics in their countries, or German or Dutch citizens who have come to fear that Italian or Greek governments are irresponsibly spending European funds for which they have to bear most of the costs. Their registration as consociated citizen provides them with the capacity to use their consociated voting rights to contest such policies and to support consociated political parties that oppose them. The horizontal expansion of voting rights guides popular dissatisfaction into productive directions. In contrast to popular initiatives and referenda, the additional statuses and voting rights do not target specific policies, but specific loci of political power in an inter- and transnational system of interdependent and joint policy-making. We believe that they will have the same consequences, however. Citizens will feel less side-lined and governments will take potential reactions beyond their national constituencies into account when they pursue policies unilaterally or interact with consociated governments.^7^

A major argument for our proposed system of transnational representation is that it may function as a wedge that drives populists and nationalists apart. New opportunities to hold political elites accountable should be attractive to populists but it’s transnational features might be a no-go or problematic in practice for nationalists. Think of an Italian party that has to make the decision whether to mobilize Italian voters to register as consociated citizens in Germany. A populist rationale would certainly speak in favour of doing so as long as the domestic discourse presents the German government as a major hindrance to European fiscal solidarity. From a nationalist point of view, the issue is much less clear-cut since such a stance is inconsistent in two respects. First, from a nationalist point of view, the entire system of transnational voting is suspect since it undermines national sovereignty. The price to give Germans a voice and vote in Rome for having a say in Berlin seems too high. Second, nationalists do not seek better representation in political centres beyond their nation state, but a return to an autarkic state of state sovereignty.

### Revitalizing political parties and party systems by transnationalizing state elections

While (some) parties and parliamentarians already consider perspectives that go beyond the narrow interests of their national electorates (Kinski and Crum, 2020), a transnational expansion of state electorates makes it much more likely that parties that do so will reap electoral rewards. This, in turn, may create incentives for other parties to do so as well. A horizontal expansion of national parliaments is likely to push parties to pay more heed to transnational problems and effects when they campaign and to present less parochial solutions. This would reduce the current gap between ‘responsive’ campaigning and ‘responsible’ governing, which, in turn, would help established parties regain voter trust and to take a stand against the populist treason and collusion rhetoric.

Overlapping electorates and transnational elections would not only help individual parties fulfill their central role as trusted intermediary institutions that link the rulers and the ruled, that is. They would also counter-act the polarization of party systems. The polarization of party systems is partly driven by populist rhetoric that accuses governing parties of betraying their constituencies when they cooperate with external actors, as well as counter-accusations that portray the support of external actors (like the Russian government) for populist parties/politicians as an effort at destabilizing democracies. A horizontal expansion of national parliaments would provide a basic legitimacy to both, domestic parties representing external interests and external actors that get involved in domestic politics, – but it would clearly specify when and to which extent it is appropriate. Involvement of external actors into domestic processes of democratic will-formation and decision-making is legitimate as far as it is based on reciprocity and legitimized through the votes of consociated citizens. In a horizontally expanded democratic order, external involvement into domestic politics is no longer dismissed as illegitimate, or based on the claims of self-authorized advocates (which is the case not only for expert bodies or non-governmental organizations, but also for transnationally oriented parliamentarians or parties). It is, instead, grounded in the will of consociated citizens and the decision-making powers of their representatives.

Domestic party systems would now function as the necessary, legitimate, pluralist and multi-directional entry points for political influence in a polycentric system that is not only increasingly characterized by cross-border flows and (inter)dependences, but also by the pooling of sovereignty and joint decision-making. This, in turn, de-fundamentalizes the conflict between parties that cater for the votes of consociated citizens and those who focus on the national constituency. The signing of a joint declaration of interdependence and identification would make it much more difficult to portray one of the two strategies as immoral or undemocratic.

In sum, political parties would not only regain their role as pluralist intermediaries between the ruled and the rulers, they would also be less driven by fear of a polarizationof the party system. This contributes to refiguring, by opening up, the processes of democratic will-formation and a less parochial process of national will-formation, without thereby questioning the integrity of national elections or the centrality of political parties that are anchored within particular states.

It is established and potentially populist (but not nationalist) parties then, or so we argue, that stand to gain most from a horizontal expansion of parliaments. We see them as the potential drivers and promoters of our proposal for this very reason. Furthermore, in contrast to earlier expansions of voting rights (e.g. towards women), our proposal is more easily sold to the constituency: for the first time, the electoral ‘in-group’ gains something through the expansion, namely the capacity to bring their electoral pressure to bear on foreign parliaments.

## Comparative advantages

In the following, we defend our proposal against alternative models of overcoming the disfigurations of democratic processes and the disruptions of transnational cooperation that are a result of technocratic intergovernmentalism. We focus on three particularly influential approaches: James Bohman’s Deliberative Transnationalism (DT), Richard Bellamy’s Republican Intergovernmentalism (RI) and, finally, Francis Cheneval’s Liberal Multilateralism (LM). DT, RI and LM represent the three most influential models for rethinking transnational democracy in contemporary normative democratic theory. Importantly, the authors we selected draw not only on three distinct, underlying normative theories of democracy. They also specify (often in cooperation with empirical scholars) particular institutional prerequisites for implementing their approaches. Like our proposal, they combine principled justifications with institutional specifications, something that we do not find very often. For each proposal, we first identify shared ground before we show how the proposed way to fulfil the need for empowered inclusion is less likely to lead to refigured processes of democratic will-formation and to efficient joint decision-making than is the case with our proposal.

### Transnationalizing will-formation *and* decision-making. Advantages compared to Deliberative Transnationalism

DT offers a reflexive approach that envisions an active role for individual citizens in transforming a system of national democracies into a transnational demoicracy. James Bohman (2007, 2010) has articulated the normative foundations and the institutional implications of such an approach most clearly. He argues that transnational politics needs to be reformed so as to achieve a ‘democratic minimum’, which consists of the citizens’ ability to ‘initiate deliberation’ across a polycentric system of government. Bohman wants to achieve ‘non-domination’ by providing citizens with deliberative opportunities across the manifold and varied institutions of a multilevel body like the EU. Bohman (2007) turns our attention to the citizens’ capacity for initiating change, even radical change, making the ‘basis of democracy *itself* the subject of the democratic deliberation of citizens’ (p. 145).

We share Bohman’s commitments to reflexivity and to individual agency. For us, this means that the concrete institutional set-up of an inter-democratic order cannot be deduced from abstract principles, but emerges out of a democratic process in which individual citizens play a central role. Bohman fails to see, however, that in a democratic order the ‘collective will’ of the citizens needs to be translated, to return to Warren (2017), into ‘decisions through which “the people” are reflexively constituted into collective agents capable of doing collective things for themselves’ (p. 43). For Bohman (2007), achieving a ‘democratic minimum’ is *not* equivalent with the ‘achievement of popular sovereignty in the sense of the final say over each and every decision’ (p. 35). This is troubling for two reasons. First, Bohman’s rather abstract idea of a human right to ‘communicative freedom’ leaves the important questions unanswered of who may decide and on the grounds of which membership(s). In other words, he provides no answer on how the third function, decision-making, of a democratic order should be fulfilled (for a similar critique: Cheneval and Schimmelfennig, 2013: 340). Second, Bohman ignores the central role that nation states play in protecting citizen’s interests and in fulfilling the promise of self-government through popular sovereignty – he gives state-peoples no role in the reflexive process of re-constituting a ‘demoicratic’ order. Similar weaknesses can be detected when we turn from principles to institutions.

His preferred model of institutions that guarantee the right to initiate deliberation is that of deliberative fora that open up the nodes of a polycentric network to citizen participation in the fashion of what has come to be known as a deliberative ‘mini public’ (Bohman, 2007: 87). As much as a ‘directly deliberative polyarchy’ (Bohman, 2007: 87) creates new venues for citizen participation, it does not fulfil the promise of popular sovereignty that lies at the centre of populist rhetoric and demands: opportunities for initiating deliberation in a transnational, multilevel apparatus like the EU are, realistically, open only to a small minority of citizens organized through unaccountable NGO’s, expert and lobby groups. Unlike parliamentary politics, what is more, deliberation in mini publics does not link processes, to turn again to Warren’s third requirement, of opinion- and will-formation to processes of decision-making. Finally, an emphasis on ‘citizen representatives’ undermines the roles of political parties as pluralist intermediary institutions.

Our approach, in contrast, stands by both the promise of enabling a reflexive transformation of the architecture of transnational rulemaking, which is so important to Bohman, and the more traditional promise of popular sovereignty through democratic representation, which Bohman mostly ignores. It does so by providing not only individual citizens but also state-peoples and important intermediary institutions like parliaments and parties an important role in shaping not only the outcomes but the very architecture of transnational rulemaking. State-peoples specify the basic architecture of the inter-democratic order when their elected representatives develop and sign ‘joint declarations of interdependence and identity’; individual citizens re-shape, in regular intervals, the electorates of participating states, when they sign ‘declarations of interest and identification’ and register as ‘consociated citizens’. In both processes, the traditional intermediary institutions of representative democracy play crucial roles. ‘Joint declarations of interdependence and identity’ are ratified in parliament and political parties help to articulate transnational policy (inter)dependencies and mobilize individual citizens to register and to vote as consociated citizens in consociated states.Our proposal, that is, draws on Warren’s crucial insight that we must not only include and empower individuals and state-peoples, but that parliaments and parties play indispensable roles in representative democracies because they operate as crucial connectors between will-formation and decision-making.

### Expanding and (not just) empowering state parliaments. Advantages compared to Republican Intergovernmentalism

Richard Bellamy’s RI represents the most clear-cut alternative to Bohman’s DT. Bellamy (2017, 2019: part 1) argues that state sovereignty is necessary for a popular sovereignty that is capable of realizing the republican value of non-domination and claims that it remains achievable and normatively warranted in an interconnected world. He develops his position by criticizing alternative approaches like federalism. Federalist ideas equate democratization of inter-democratic orders with strengthening supranational parliaments (like the EU parliament), a solution that suffers from three weaknesses. First, it does not pay sufficient heed to the socio-economic as well as cultural-linguistic diversity among EU member states and the salience of divergent national interests and perspectives. A supranational parliament runs the risk of creating consistent and isolated segmental minorities. Second, the lack of a thick common identity and the corresponding thinner bonds among citizens limits the kind of policies that can be pursued. Third, not only the horizontal bonds among the citizens, but also the vertical links between the represented and the representatives are much thinner than on a national level. The constituencies of EU parliamentarians are much larger than those of national parliamentarians, trans-European interest groups and parties barely exist and European Parliament (EP) elections consist of second-order national elections.

Bellamy’s alternative solution lies in the demand that parliaments must complement governments as representatives of sovereign state-peoples in such a way that they reduce the domination of the executive branch in the domestic realm and at the same time show equal concern and respect to other state-peoples. In order to reach the first goal, parliamentarian oversight over governments has to be strengthened.^8^ Such a strengthening of the legislature in the domestic realm reduces not only the dominance of the executive branch, but should also secure a recognition of national peculiarities in the international realm. To advance inter-national recognition of this kind, Bellamy argues for stronger inter-parliamentarian collaboration, which he hopes will create a joint public discourse that stimulates common sympathies and helps to uncover joint interests, or, as Bellamy puts it, an ‘ontology of civicity’ (Bellamy, 2019: 97–130).^9^

We share some of Bellamy’s scepticism towards federalist approaches, although we would put less emphasis on the missing thickness of horizontal bonds among different state-peoples. From the perspective of our approach, which focuses on the political foundations of a democratic order, what is most problematic are the thinner links that the represented have to their representatives on a supranational level compared to the national level, since the weak and nationally oriented intermediary institutions (media, interest organizations and political parties) that exist on the trans- and supranational level contribute greatly to the current disfiguration of processes of will-formation. Furthermore, we agree with Bellamy that we need to strengthen the role of national parliaments in international affairs. In contrast to him, we argue that this is only productive if state parliaments are transnationalized at the same time.

In respect to the first function of democracy – empowered inclusion – RI mirrors DT’s weakness. Whereas the latter puts citizens centre stage and ignores the fundamental role that state-peoples have to play in a demoicratic order, RI recognizes only state-peoples and dismisses citizens as principals of an inter-democratic union. This leads to solutions which emphasize inter-institutional collaboration and ignore individual agency. Strengthening the legislative branch vis-à-vis the executive branch of government in international affairs is certainly a first step towards reducing technocratic and plebiscitarian disfigurations of democratic will-formation, since it undermines the gate-keeper position that governments currently occupy between the domestic and international realm. Problematically, however, this strategy is caught on two horns of a dilemma. Depending on whether national parliaments decide to focus on strengthening transnational problem-solving capacities and inter-parliamentary cooperation or on defending the national interest in international negotiations, RI either alienates parliaments from their constituencies or blocks international cooperation by strengthening the alignment of popular sovereignty and a politics of nationalism.

On one horn of the dilemma, RI’s call for ‘domesticating’ international policy-making by giving national parliaments a greater say in matters of transnational politics (Kröger and Bellamy, 2016: 139) and opening venues for cross-parliamentary cooperation is unlikely to assuage the populists’ worry about a lack of popular sovereignty. There is a high risk that the opposite might be the case, namely that the RI strategy alienates national parliaments from their constituencies. The high institutional complexity involved (Auel and Neuhold, 2017), and the time invested into inter-institutional activity – time missing from interacting with their constituency (De Wilde and Raunio, 2018) – are likely to reinforce a view of national parliaments as out of touch with the common people, a picture pushed by populist rhetoricians. What is more, the focus on inter-parliamentary cooperation as the bedrock of international cooperation is particularly problematic against the backdrop of a development towards the ‘cartelization’ of traditional parties and the populist reactions this cartelization has arguably produced. The more parliaments want to be able to effectively solve international problems through inter-parliamentary cooperation the more the political parties that make up these parliaments need to find common ground. The more this is the case, the more the possibility for politicizing international politics along ideological (e.g. left–right) party lines is curtailed (Sprungk, 2016).

On the other horn of the dilemma, the RI strategy, which assigns to national parliaments the role of enacting popular sovereignty and representing the interest of a particular people, risks reinforcing the alignment between populism and nationalism. As long as state parliaments are accountable only to their national constituency, the empowerment of such parliaments is likely to provide parties with an incentive to engage in parochial electoral campaigns. To provide state parliaments with greater capacities for oversight of governmental activities in international affairs increases the likelihood that the space for compromise in international negotiations shrinks. The institutional proposals made by RI risks, that is, to undermine the effectiveness (and fairness) of joint decision-making.^10^

Our proposal towards a horizontal expansion of national parliaments escapes this dilemma. Under a regime of horizontally expanded parliaments parties gain electoral incentives for looking beyond the narrow margins of national interest without abandoning their role as agents of domestic popular sovereignty. Importantly, parties under this regime face less pressure to move towards common ground to better enable cross-parliamentary cooperation. Rather, parties will take the expanded electorate into account in their electoral campaigns and politicize issues of transnational interest along ideological party lines. Finally, where RI risks to reinforce the alignment between populism and nationalism, horizontal expansion may act as a wedge between populists who are likely to grasp the opportunity for a transnational expansion of popular sovereignty and nationalists who want to realign the demos with the sovereign nation state.

### Recognizing mobile *and* sedentary citizens in the *domestic* and (not just) in the international realm. Advantages compared to Liberal Multilateralism

In contrast to the advocates of DT and RI, recent proponents of LM share our conviction that democratic systems of transnational rulemaking must include and empower two distinct kind of actors: individual citizens and state-peoples. Liberal multilateralists like Cheneval are normative individualists, and partly follow (primarily in their methodological approach) John Rawls (1999) who claims in his *Law of Peoples* that individuals can be free and equal only as members of a sovereign state that guarantees the rule of law, individual freedoms and a responsive and accountable government. Unlike Rawls, however, Cheneval points to the fact that the interests and values of individual citizens

need not necessarily coincide with the interests and common purposes of their respective statespeople. Free citizens of a liberal state might engage in transnational associations with purposes that differ from the collective interests of its people as represented by the government. (Cheneval, 2008: 42)

Drawing on this insight, Cheneval’s LM represents a fundamentally pluralist stance based on the claim that neither state-peoples nor individual persons can be reduced to each other in inter-democratic relations. Both kinds of principals must be separately recognized, included and empowered.

This basic agreement notwithstanding, we disagree with LM in two important respects: its prioritization of mobile actors (rather than empowering mobile *and* sedentary actors) and its institutional strategy of fulfilling the function of empowered inclusion on a supranational level, which we discuss in this order.

Proponents of LM demand of states forming a democratic union, first, a mutual recognition of the equal legitimacy of the regulations of other member states. They demand, second, that they refrain from discriminating against the citizens and corporations of other member states and, third, that they grant mobile citizens not only the right to exit and entry, but also an inclusion into the national demos (Cheneval, 2008: 49–53; Cheneval et al., 2015: 7–14). The demand to the reciprocal recognition of competing regulations is, effectively, an expression of a ‘negative integration’ strategy, which de-regulates the transnational realm by stimulating regulatory competition (Scharpf, 1999). De-regulation, which empowers mobile private actors like multinational corporations and holders of high levels of human and financial capital, pours oil on the flames of a populist rhetoric that depicts the world as one which sees transnational elites collude and profit against ‘the people’. The same is true of the one-sided liberal emphasis on constitutional rights against discrimination and on comprehensive rights for migrants, which deepens an existing cleavage between mobile and sedentary citizens (Goodhart, 2017).

In contrast, our proposal seeks to internalize the external effects of regulatory competition: those who are systematically affected by policies of individual states and subjected to intergovernmental rulemaking get a say in formulating state policies (in the former case) and state positions (in the latter case). It also seeks to balance the interests of mobile citizens and the interests of sedentary citizens in all involved states. Consociated citizens can influence alien regulation that systematically affects their interests and joint regulations that they are subjected to, through their voting rights in consociated nation states. The crucial difference to LM is that debates on the adequacy of regulatory policies and on transnational discrimination take place in the horizontally expanded law-making process within multiple nation states and not only in supranational courts. Furthermore, our proposal grants political rights in multiple member states not merely to mobile citizens and to those with multiple citizenships but to sedentary mono-citizens as well. The institutional logic of LM effectively equates state-peoples with a national community of sedentary mono-citizens, whereas the empowered inclusion of individuals is directed at mobile agents with transnational links. Empowered inclusion, as we want to stress, must mean equal inclusion of mobile *and* sedentary agents by granting all consociated citizens voting rights in horizontally expanded parliaments. Only an empowered inclusion of this kind can provide an effective response to the growing political polarization between the ‘anywheres’ and the ‘somewheres’ (Goodhart, 2017).

The second central difference to LM has to do with the level on which both state-peoples and citizens are included in transnational processes of will-formation and decision-making. Proponents of LM want to push for a solidification of the vertical multi-level solution that has already taken hold in the EU (Cheneval and Schimmelfennig, 2013). They argue that state-peoples and citizens must not only be represented equally through distinct institutions, but that they must have equal legislative rights (Cheneval and Schimmelfennig, 2013: 343). Despite the fact that LM is based on the ‘demoicratic’ presumption that there exist no consolidated demoi beyond national ones, and despite the fact that they want to avoid crossing ‘the Rubicon’ (Nicolaidis, 2013) that separates a confederation (individual units dominate) from a federation (the joint union dominates), their main pathway for overcoming technocratic intergovernmentalism and for the inclusion of individual citizens is the empowerment and transnationalization of the European parliament. In respect to empowerment, the EU has already made major steps towards this solution, inasmuch as ‘co-decision’ – the procedure in which the European Parliament, representing European citizens, and the European Council, representing European state-peoples, must find a consensus – has become the ordinary legislative procedure (Cheneval and Schimmelfennig, 2013: 345). When it comes to the transnationalization of the EP, this is not yet the case. Proponents of LM generally accept the current situation in which European parliamentarians are elected on national lists representing national constituencies; but they support the idea that a subgroup of EU parliamentarians should be elected on the basis of transnational lists in a pan-European district, representing a European demos (Scherz, 2017). Yet, attempts to use the Brexit for implementing this idea failed (Bartl, 2020).

The main problem with proposals of this kind is that they necessarily involve a transfer of competences and power from national governments to a supra-national (European) parliament. In other words, LM proposes not merely a shift of competences from the executive to the legislative branch of government (which we support) but a more problematic shift from national to European constituencies and institutions. The twofold resistance this produces, from the executive on the one hand, and from national legislatures and nationally anchored representatives on the other, seems to block any viable pathway towards a realization of such proposals (e.g. an important argument against transnational lists was the fear that those elected through national lists would be ‘lesser’ EU parliamentarians, Bartl, 2020: 60). We argue that our horizontal expansion of national parliaments need not fear a similar kind of twofold resistance. It aims to strengthen national parliaments only – and it accepts that ‘consociated representatives’ are complementary, and in this sense ‘secondary’ representatives.^11^

What is more, our proposal contains a different vision of how a transnationalized party system emerges. LM builds on existing federations of political parties and on the party caucuses in the EP. Whereas the former tends to be little more than an ‘ideationally delocalized’ (White and Ypi, 2016: 196) cooperation of political elites, the latter are bound together by the institutional logic of forming a joint parliamentarian position vis-a-vis the Commission and the Council. Within our approach, in contrast, the formation of transnational party alliances will be stimulated primarily by the aim to support a domestically anchored party in gaining votes from consociated citizens. In consequence, the core actors are still national parties which help each other based on the principle of reciprocity and which do so in their communication to the citizens.^12^

We should note, finally, that some defenders of LM, for example, Francis Cheneval, have come to acknowledge the pitfalls of strengthening the EU parliament, and, mirroring RI, have come to rely more heavily on the idea of giving national parliaments a greater capacity to influence European legislation (Cheneval et al., 2015: 7) – although compared to RI, LM puts less emphasis on horizontal collaboration among national parliaments and more on vertical collaboration between national parliaments and the EU parliament.^13^ The proposals of Cheneval et al. face the same problem as those pushed by Richard Bellamy; however, collaboration among different kinds of representatives is still readily portrayable as elite collusion and might not strengthen but weaken the links between representatives and the represented. Our proposal conceptualizes the way in which national representatives get involved in transnational relations differently: not by collaboration among representatives of different kinds of institutions and constituencies, whose collaboration is always overshadowed by an inherent competition for competences, but by extending established representative institutions and constituencies on the national level.

## Conclusion

We believe that representative democracies can tackle the intertwined challenges of technocratic (and plebiscitarian) intergovernmentalism and populist nationalism simultaneously. We propose that they jointly constitutionalize and expand the system of horizontally overlapping state demoi/electorates that currently emerge almost unnoticed as an unintended side-effect of the spread of multiple citizenships and non-resident voting (Michel and Blatter, 2021). An empowered inclusion of consociated citizens into the electorates of states provides the foundation for a mutual opening of democratic processes of collective will-formation and decision-making, anchored within the established institutional structures and democratic procedures of nation states. This would address the elitist bend of intergovernmentalism by bringing international policy-making closer to the people and revitalize political parties and national parliaments as central intermediary institutions within a pluralist democracy. Our proposal takes seriously the concerns that populists have raised against the technocratic character of international policy-making, but it provides decidedly non-populist answers to these concerns. The same is true of nationalism. While we recognize state-peoples and states as cornerstones of democratic self-determination, we provide solutions that help overcome the narrow national character of core institutions of collective will-formation and decision-making within states. The simultaneous transnationalization of state-peoples, parties and parliaments would not only help to reintegrate and to revitalize the core institutions and processes of representative democracies. It would also make it easier for liberal representative democracies to address the current challenges of a globalized world cooperatively and effectively, bringing them into a better position for confronting authoritarian temptations from within and authoritarian competitors from abroad.

## Notes

1. Whereas theorists prefer the term ‘demoi’, empiricists prefer ‘electorates’. Since the aim of this paper is to speak to both, we apply both terms synonymously.

2. The proposal in its full institutional complexity has been presented and debated in earlier publications (Blatter, 2018; Blatter and Bauböck, 2019). The main goal in this publication is to anchor it in democratic theory and contrast it to principled alternatives.

3. Negative integration leads to a situation in which citizens of State A are ‘systematically affected’ by the policies of State B (e.g. lower environmental regulations), since they may not shield themselves against the negative external effects of these policies by introducing policies that discriminate against the products produced under such regulations. ‘Systematically affected’ does not correspond to the ‘all affected interest principle’ since it refers not to individual policy decisions, but to a general constellation between the citizens of a state and the government of another state, which is created by negative integration; and it differs from the ‘all subjected to law’ principle, since the citizens of State A are not legally subjected to the policies of State B (for an encompassing debate on these principles of democratic inclusion, see Bauböck, 2018).

4. For a discussion on the meaning of ‘responsive’ versus ‘responsible’ governing, see, e.g. Bardi et al., 2014.

5. The term ‘consociation’ alludes to the notion of ‘consociational democracy’, a concept that Arend Lijphart (1969) developed in order to describe and explain how a democracy can function in a stable way in states where no strong integrated culture or identity exists. We want to emphasize, however, that our proposal goes beyond Lijphart’s focus on the cooperative behaviour of elites who represent separated cultural groupings. In many respects, our proposal is closer to what has been called a ‘centripetal approach to democracy’ (e.g. Lacey, 2017), which seeks to address the problem of a strongly fragmented demos by introducing voting systems that incentivise political parties rooted in one group to reach out to potential voters in other groups.

6. For example, as many as 25 percent of all Swiss are currently dual citizens; among the Swiss abroad, 75 percent are dual citizens. Since Switzerland grants external nationals voting rights in national elections, they can vote in all countries of nationality (Blatter, 2018). Switzerland might be a frontrunner in the development of overlapping memberships, but for both preconditions for overlapping demoi, we observe world-wide trends. For the acceptance of dual citizenship, see Vink et al., 2019, for the trend to grant non-resident national voting rights on the state level, see Arrighi and Bauböck, 2017.

7. Note, however, that the proposal does not presume a coherent perspective or interest of consociated citizens, and refigures democratic will-formation by acknowledging and strengthening political pluralism. To build on our example, a majority of Italians who register in Germany might vote for German parties who support European fiscal solidarity, but it is also not unlikely that Italians who prefer a less lenient fiscal policy in Italy would register and vote in the German election in order to make sure that the German government keeps its pressure up.

8. Practices in many countries are in line with this approach. For an overview: Rozenberg and Hefftler, 2015. For a differentiated recent analysis, see e.g. Winzen et al., 2018.

9. This part of the proposal has already been put into action: See, for example, the agreement that the German and the French Parliaments recently signed to establish a joint parliamentary assembly, not just in order to strengthen and to democratize their bilateral relations, but also to foster convergence on national positions on the European level. https://www.bundestag.de/resource/blob/577996/a32cc8d55b73523c2cceb64ddcf9c5be/kw46_dt_frz_parlamentsabkommen_entwurfstext-data.pdf

10. Proponents of RI realize that strengthening the oversight roles of national parliaments carries the risk of hampering joint decision-making. As a remedy, they propose inter-institutional instruments like a *Parliamentary Legislative Initiative*, which would allow a plurality of state parliaments not only to block, but also to simulate international regulation (Kröger and Bellamy, 2016: 147–148). However, parliamentarians would face an even larger trade-off between investing in international cooperation and investing in their links to voters.

11. According to our proposal, each citizen has primary representatives in her or his national parliament, plus further representatives in parliaments of consociated states. The latter are ‘secondary’ not only because it is not a substitute, but only complement to the first, but also because their number is limited, and does not correspond to the number of eligible voters (see Blatter, 2018: 22–27, 2019: 51–52).

12. The idea to transnationalize the elections to the EP by nominating ‘*Spitzenkandidaten*’ has failed; first, national parties did not emphasize their European *Spitzenkandidat* (Braun and Popa, 2018); second, after the 2018 election, the head of governments ignored the electoral result.

13. In 2017, a group of mainly French academics (including Thomas Piketty) joined Benoît Hamon, the presidential candidate of the French Socialist Party, in pushing for the creation of a Euro-zone Parliamentary Assembly, which would mostly include members of national parliaments but also members of the European Parliament, for the sake of democratizing and supranationalising political decision-making within the Treaty on Stability, Coordination and Steering in the Economic and Monetary Union (see: http://tdem.eu/en/treaty/; 1.11. 2021)
